# Apoptosis of Bone Marrow Mesenchymal Stem Cells Caused by Homocysteine via Activating JNK Signal

**DOI:** 10.1371/journal.pone.0063561

**Published:** 2013-05-07

**Authors:** Benzhi Cai, Xingda Li, Yang Wang, Yanju Liu, Fan Yang, Hongyang Chen, Kun Yin, Xueying Tan, Jiuxin Zhu, Zhenwei Pan, Baoqiu Wang, Yanjie Lu

**Affiliations:** 1 Department of Pharmacology, State-Province Key Laboratories of Biomedicine- Pharmaceutics of China, Harbin Medical University, Harbin, Heilongjiang Province, China; 2 Institute of Cardiovascular Research, Harbin Medical University, Harbin, Heilongjiang Province, China; University of Udine, Italy

## Abstract

Bone marrow mesenchymal stem cells (BMSCs) are capable of homing to and repair damaged myocardial tissues. Apoptosis of BMSCs in response to various pathological stimuli leads to the attenuation of healing ability of BMSCs. Plenty of evidence has shown that elevated homocysteine level is a novel independent risk factor of cardiovascular diseases. The present study was aimed to investigate whether homocysteine may induce apoptosis of BMSCs and its underlying mechanisms. Here we uncovered that homocysteine significantly inhibited the cellular viability of BMSCs. Furthermore, TUNEL, AO/EB, Hoechst 333342 and Live/Death staining demonstrated the apoptotic morphological appearance of BMSCs after homocysteine treatment. A distinct increase of ROS level was also observed in homocysteine-treated BMSCs. The blockage of ROS by DMTU and NAC prevented the apoptosis of BMSCs induced by homocysteine, indicating ROS was involved in the apoptosis of BMSCs. Moreover, homocysteine also caused the depolarization of mitochondrial membrane potential of BMSCs. Furthermore, apoptotic appearance and mitochondrial membrane potential depolarization in homocysteine-treated BMSCs was significantly reversed by JNK inhibitor but not p38 MAPK and ERK inhibitors. Western blot also confirmed that p-JNK was significantly activated after exposing BMSCs to homocysteine. Homocysteine treatment caused a significant reduction of BMSCs-secreted VEGF and IGF-1 in the culture medium. Collectively, elevated homocysteine induced the apoptosis of BMSCs via ROS-induced the activation of JNK signal, which provides more insight into the molecular mechanisms of hyperhomocysteinemia-related cardiovascular diseases.

## Introduction

Over the past decades, cardiovascular diseases remain a leading cause of mortality all over the word. Though the therapeutic advances have improved the survival of patients with cardiovascular diseases in clinics, the loss of cardiac cells due to apoptosis or necrosis in injured hearts can not be reversed. Bone marrow mesenchymal stem cells (BMSCs) have emerged as a novel therapeutic approach for cardiovascular diseases. BMSCs are found in the bone marrow, adipocytes, cord blood, peripheral blood, and fetal liver and lung [1], and have previously been regarded to play only a supportive role in hematopoietic homeostasis in bone marrow by secreting hematopoietic cytokines [2], [3]. Lately, increasing evidence uncovered that BMSCs are capable to differentiate into multiple cell lineages such as cardiomyocytes and endothelial cells [4]. Especially, after stimulated by inflammatory and cytokines such as stromal cell-derived factor-1 (SDF-1), BMSCs was shown to enter the circulating blood and then migrate to the injured hearts [5], [6], which enable BMSCs to regenerate the myocardium by transdifferentiation, neovascularization and paracrine actions [7]–[9]. Nevertheless, some pathological stimuli such as hypoxia, ischemia, inflammation or acidosis commonly led to the dysfunction or apoptosis of BMSCs, which servers as a new reason of cardiovascular conditions [9]–[11]. Several studies have displayed only modest or even low levels of local retention, survival, and differentiation of BMSCs into cardiac cells under ischemic and inflammatory injury [12]–[15]. On the contrary, preconditioning of BMSCs with hypoxia or some chemicals enhanced its resistance to these damaged factors and protected BMSCs against apoptosis [15], [16].

As a novel important independent risk factor for cardiovascular diseases, hyperhomocysteinemia is strongly associated with coronary heart disease, heart infarction, stroke, atherothrombosis, peripheral vascular disease, etc [17], [18]. Elevated plasma homocysteine level induces apoptosis of cardiomyocytes, promotes proliferation of endothelial cells and activates inflammatory cells [19]–[21]. Even though a large body of experimental studies demonstrated that hyperhomocystemia is a new pathogen of cardiovascular diseases, but there is, so far, no evidence of the effects of elevated homocysteine level on the proliferation and apoptosis of rat BMSCs. The present study was aimed to investigate the proapoptotic actions of homocysteine on BMSCs and explore its potential mechanisms.

## Materials and Methods

### Ethics Statement

All the protocols in the present study have been approved by the Animal Care and Use Committee (IACUC) of Harbin Medical University. All the procedures were in compliance with the National Institute of Health Guide for the Care and Use of Laboratory Animals (NIH Publications No. 80–23). In this study, homocysteine (Sigma, USA) was made fresh the day of the experiment by diluting with distilled water.

### Bone Marrow Mesenchymal Stem Cells

The method to isolate and culture BMSCs were just as previously described [22]. After anesthesia, the femurs and tibias were taken from immature Sprague–Dawley rats (weighing 100±20 g) and bone marrow cells were collected from the bone marrow and then transferred into culture flasks with culture medium special for Mesenchymal Stem Cells (Stem Cell Technologies Inc.) supplemented with penicillin (100 U/ml)/streptomycin (100 U/ml) at 37°C with 5% CO_2_. Three days later, the culture medium was changed, and then the cells in the flasks were passaged at 1:2 ratio when reaching 80% confluence. All experiments in this study were performed using cells of the 3rd passage.

### MTT Assay

Cellular viability was assessed by MTT assay just as described previously [23] with some modifications. In brief, after exposing to different concentrations of homocysteine for 24 h, the cells were further incubated with the MTT reagent for 4 h at 37°C with 5% CO_2_. Then, DMSO 1 ml was added to dissolve farmazan crystals and the OD values were taken at 490 nm by using an Elisa plate reader.

### AO/EB Staining

Acridine orange/ethidium bromide (AO/EB) double staining was used to detect the apoptosis of BMSCs as described previously [23].

### Hoechest 333342 Staining

BMSCs were fixed with 4% paraformaldehyde for 30 min at room temperature. Then, the cells were stained with Hoechst 333342 for 20 min. After washing twice with serum-free DMEM, the cells were resuspended in serum-free DMEM for morphological observation using the fluorescence microscope.

### Live/Dead Staining

LIVE/DEAD® Viability/Cytotoxicity Assay Kit (Invitrogen, USA) was used to observe live and dead cells. In brief, BMSCs were plated on coverslips and then were treated with different concentrations of homocysteine. The cells were then washed with PBS and stained according to manufacturer’s instructions. BMSCs were photographed under a fluorescence microscope. The stained live cells display green fluorescence and stained dead cells display red fluorescence.

### TUNEL Assay

Terminal deoxynucleotidyl transferase dUTP nick end labeling (TUNEL) assay was used to detect the proapoptotic effects of homocysteine on BMSCs. The method to perform TUNEL assay is just was described previously [23]. BMSCs were fixed with 4% paraformaldehyde solution for 1 h at room temperature, and then permeabilized in 0.1%Triton X-100, followed by freshly prepared TUNEL reaction mixture for 1 h in a dark room. The coverslips were then washed with PBS and observed under a fluorescence microscope.

### Measurement of Reactive Oxygen Species

Intracellular ROS level of BMSCs was quantified by ROS Detection Assay Kit (Beyotime, China). BMSCs were collected and exposed to 10 µM DCFH-DA for 20 min at 37°C in a dark room. After that, BMSCs were washed twice and were then photographed under a fluorescence microscope.

### Mitochondrial Membrane Potential

Mitochondrial membrane potential was determined using JC-1 probe (Beyotime, China). Briefly, after treatment with homocysteine for 24 h, BMSCs were stained with 10 µM of JC-1 for 20 min at 37°C. After washing twice with buffer solution, BMSCs were analyzed by using a fluorescence microscope.

### ELISA Assay

The procedure to measure VEGF and IGF-1 concentration in the culture medium of BMSCs was just as described below. In brief, after BMSCs were treated by homocysteine 30, 100, 300 and 1000 µM for 72 h, the cultured medium was collected and then centrifuged at 3000 g for 10 minutes. The VEGF and IGF-1 concentration in the supernatants was assayed using VEGF and IGF-1 ELISA kits (WuHan Boster Co., Ltd., China) according to the manufacturer’s instructions. The experiment was performed three times.

### Western Blot

Protein samples were extracted from cultured BMSCs after treatment with homocysteine. Protein concentration was determined using the BCA method as recommended by the manufacturer. After boiled for 5 min, the protein samples were fractionated by SDS-PAGE (10%–15% polyacrylamide gels) and transferred to PVDF membrane (Millipore, Bedford, MA). The membranes were blocked with milk powder for 1 h at room temperature, and then incubated with primary antibody for phospho-JNK (Abcam, UK), JNK (Abcam, UK), phospho-p38 MAPK (Abcam, UK), p38 MAPK (Abcam, UK), phospho- ERK1/2 (Abcam, UK), ERK1/2 (Abcam, UK), phospho-p53 (Santa Cruz, USA), caspase-3 (Santa Cruz, USA), cleaved caspase-3 (Santa Cruz, USA), Bcl-2 (Santa Cruz, USA) at 4°C overnight. After washing three times, the membranes were incubated with mouse or rabbit secondary antibodies for 1 h at room temperature. Western blot bands were quantified using Odyssey v1.2 software by measuring the band intensity (area×OD) for each group and normalizing to GAPDH (Santa Cruz, USA) as an internal control.

### Statistical Analysis

All experimental data were presented as the mean ± SEM. ANOVA or t-test was used to compare mean values using GraphPad Prism software. Values of p<0.05 were considered statistically significant.

## Results

### Effects of Homocysteine on the Morphology and Viability of BMSCs

**Figure 1:**
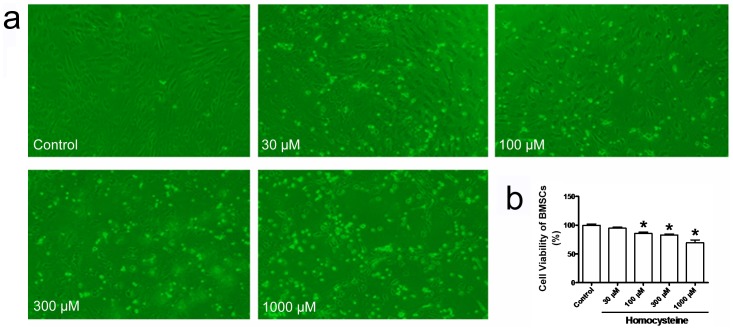
Effects of difference concentrations of homocysteine on the morphological appearance and cellular viability of BMSCs. (a) The morphology of cultured BMSCs was observed after treatment with homocysteine 30, 100, 300 and 1000 µM for 24 h. Homocysteine caused aberrant morphological appearance of BMSCs. (b) Homocysteine significantly decreased the cellular viability of BMSCs in a concentration-dependent manner. * p<0.05 versus Control.

Firstly, we determine if homocysteine can result in the morphological changes of BMSCs. As shown in Figure 1a, exposure of BMSCs to homocysteine 100, 300 and 1000 µM for 24 h caused obvious cellular morphological changes such as cellular shrinkage. Then, the influence of homocysteine on the cellular viability of BMSCs was assessed by MTT assay. As illuminated in Figure 1b, pretreatment with homocysteine 100, 300 and 1000 µM for 24 h exerted remarkably inhibitory effects on the cellular viability of BMSCs (p<0.05). The cellular viability of BMSCs were significantly decreased by homocysteine 100, 300 and 1000 µM to 85.59±4.69%, 82.82±4.08% and 69.27±9.97 after treatment for 24 h, respectively, but it was not altered by homocysteine 30 µM after treatment for 24 h (Figure 1b). Though the cellular viability of BMSCs was decreased by homocysteine, MTT can not represent the apoptosis of BMSCs induced by homocysteine. Thus, in order to confirm that homocysteine causes BMSCs apoptosis, AO/EB, Hoechest33342 and Live/Death staining were employed in this study.

### Homocysteine Induced the Apoptosis of BMSCs

**Figure 2:**
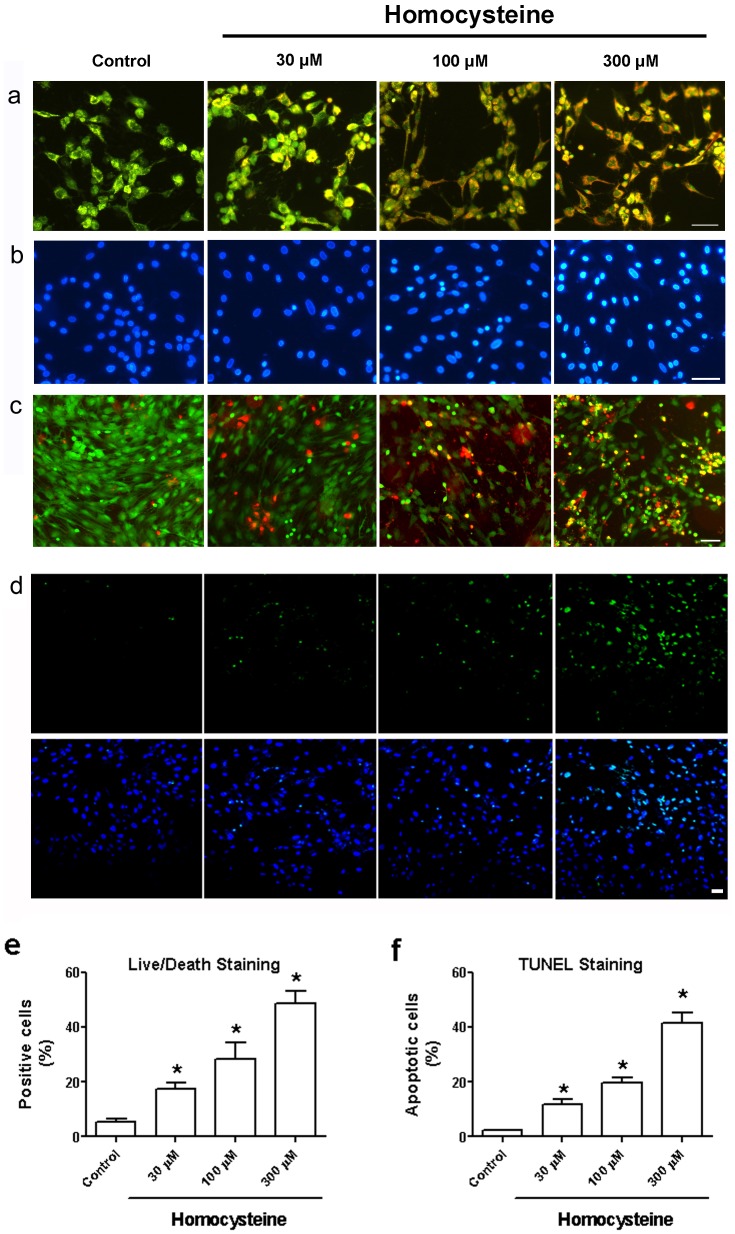
Homocysteine induced apoptotic cellular changes of BMSCs. (a) AO/EB double staining demonstrated the effects of difference concentrations of homocysteine on the apoptosis of BMSCs. BMSCs were incubated with homocysteine for 24 h. (b) Hoeschest33342 staining detected the changes in the nucleus of BMSCs after treatment with homocysteine 30, 100 and 300 µM (scale bar, 20 µm). (c, e) The inhibitory effects of homocysteine on BMSCs were determined by Live/Dead staining. (d, f) TUNEL was used to determine the effects of homocysteine on BMSCs apoptosis (n = 3 independent experiments). * p<0.05 versus Control.

As displayed in Figure 2a, AO/EB double staining demonstrated that treatment with homocysteine 100 and 300 µM for 24 h induced apoptosis of BMSCs characterized by the distinctive red-orange fluorescence. Hoechest33342 staining also showed that BMSCs after exposing to different concentrations of homocysteine for 24 h displayed apoptotic morphological changes such as nucleus condensation (Figure 2b). Likewise, Live/Dead staining also showed that the percentage of staining-positive BMSCs was significantly increased from 5.5% to 28.3% and 48.7% after incubation with homocysteine 100 and 300 µM for 24 h, respectively (Figure 2c, e). We also performed TUNEL assay to observe if homocysteine induced BMSCs apoptosis. As shown in Figure 2d, treatment with homocysteine 100 and 30 0µM for 24 h increased the positive apoptotic cell percentage from 2.3% to 19.8% and 41.4% in BMSCs, respectively (p<0.05, n = 3 independent experiments, Figure 2f). These studies suggest that homocysteine plays a proapoptotic role in BMSCs.

### Homocysteine Enhanced ROS and Depolarized Mitochondrial Membrane Potential of BMSCs

**Figure 3:**
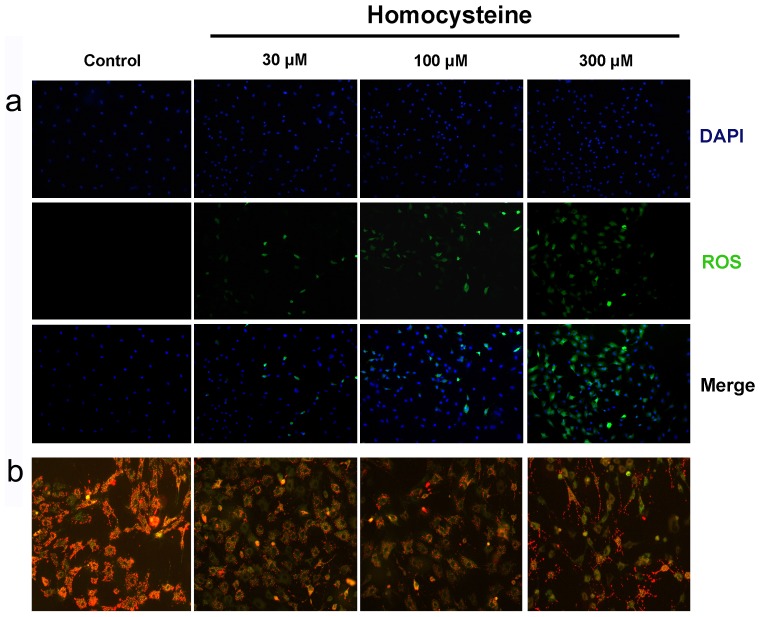
Effects of homocysteine on intracellular ROS and mitochondrial membrane potential of BMSCs. (a) Intracellular ROS level was measured in BMSCs treated with homocysteine 30, 100 and 300 µM for 24 h by DCFH-DA staining. The ROS level was gradually increased with the increase of homocysteine concentration. (b) Homocysteine induced an obvious depolarization of mitochondrial membrane potential in BMSCs apoptosis by JC-1 staining.

It is well documented that reactive oxygen species (ROS) is involved in apoptosis of many cell types [24]. Oxidative stresses caused by ROS are shown to initiate or promote apoptosis via oxidizing mitochondrial membrane phospholipids and depolarizing mitochondrial membrane potential which produces more ROS [24], [25]. We therefore investigated the influences of homocysteine on the production of ROS and mitochondrial membrane potential by DCFH-DA staining and JC-1 staining, respectively. As shown in Figure 3a, DCFH-DA staining showed that both the intensity of green inflorescence and the percentage of ROS-positive cells were significantly increased in the presence of homocysteine 300 µM for 24 h. Moreover, treatment of BMSCs with homocysteine for 24 h was able to cause the obvious depolarization of mitochondrial membrane potential (Figure 3b). These indicate that ROS-mediated mitochondrial dysfunction is involved in homocysteine-induced BMSCs apoptosis.

### ROS was Involved in Homocysteine-induced Apoptosis of BMSCs

**Figure 4:**
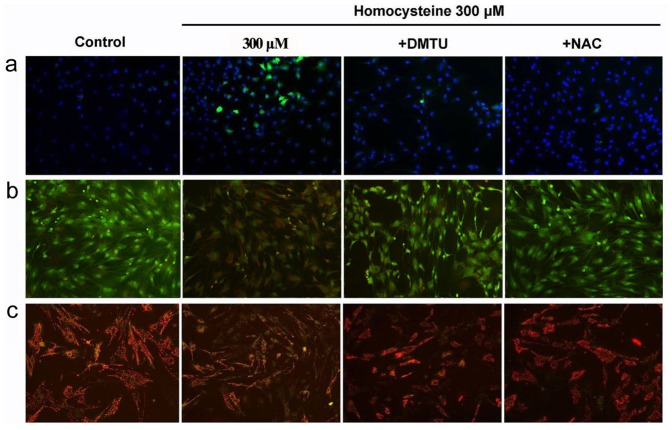
Increased ROS is required for homocysteine-induced apoptosis of BMSCs. (a) DMTU and NAC attenuated the increase of ROS level by homocysteine in BMSCs. (b) Effects of DMTU and NAC on the apoptotic appearance of BMSCs. The inhibition of ROS with DMTU and NAC abolished the apoptosis of BMSCs induced by homocysteine. (c) Homocysteine-induced depolarization of mitochondrial membrane potential was also effectively reserved by DMTU and NAC in BMSCs.

To confirm whether ROS is required for homocysteine-induced apoptosis of BMSCs, we employed two specific antioxidants DMTU and NAC. As displayed in Figure 4a, the increase of ROS in BMSCs was obviously increased by homocysteine 300 µM after treatment for 24 h, which can be effectively reversed by individual pretreatment with DMTU and NAC. AO/EB double staining also showed that DMTU and NAC can reverse homocysteine-induced apoptosis of BMSCs (Figure 4b). Moreover, the depolarization of mitochondrial membrane potential induced by homocysteine was effectively reserved after pretreatment with DMTU and NAC for 24 h, indicating ROS-mediated mitochondrial membrane depolarization takes part in homocysteine-induced the impairment of BMSCs (Figure 4c).

### Effects of MAPK Inhibitors on Homocysteine-induced Apoptosis of BMSCs

**Figure 5:**
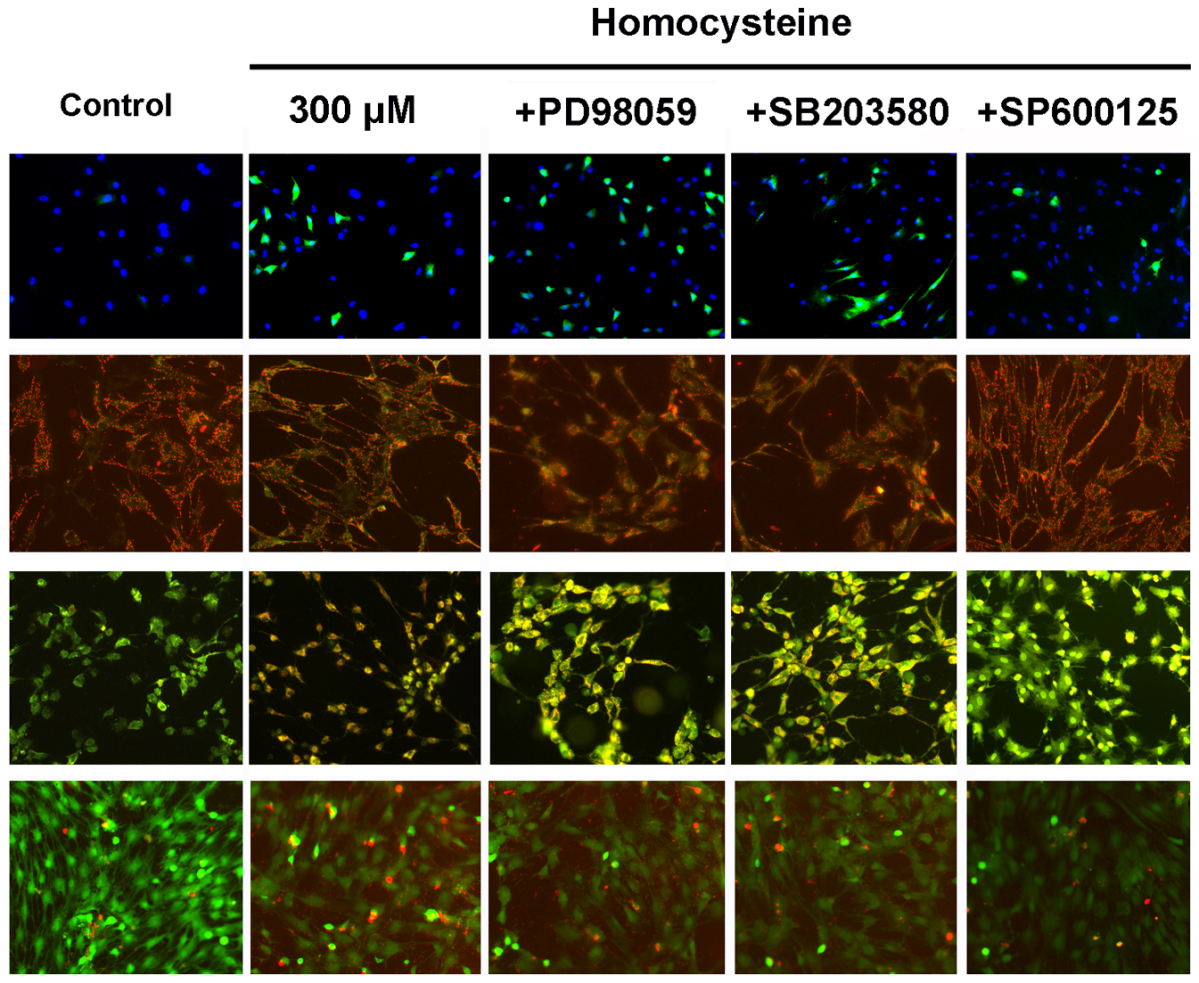
JNK signal is involved in the apoptosis of BMSCs induced by homocysteine. JNK specific inhibitor effectively attenuated the apoptosis induced by homocysteine 300 µM in BMSCs. However, p38 and ERK specific inhibitors did not affect homocysteine-induced apoptotic morphological changes in BMSCs.

A large body of evidence has shown that MAPK signal pathway is involved in ROS-mediated cellular apoptosis [26]. However, whether MAPK signal pathway also plays a critical role in homocysteine-induced BMSCs apoptosis remain unknown. Here, we found that the specific JNK inhibitor, SP600125 (10 µM) could reverse homocysteine-induced BMSCs apoptosis featured by the inhibition of mitochondrial membrane potential depolarization and nucleus damage, without the impact on intracellular ROS level (Figure 5). Neither p38 MAKP inhibitor SB203580 (5 µM) nor ERK inhibitor PD98059 (25 µM) is able to reverse homocysteine-induced apoptotic morphological changes. These results indicate that JNK signal pathway is required for homocysteine-induced BMSCs apoptosis.

### Homocysteine Induced Activation of JNK Signal in BMSCs

**Figure 6:**
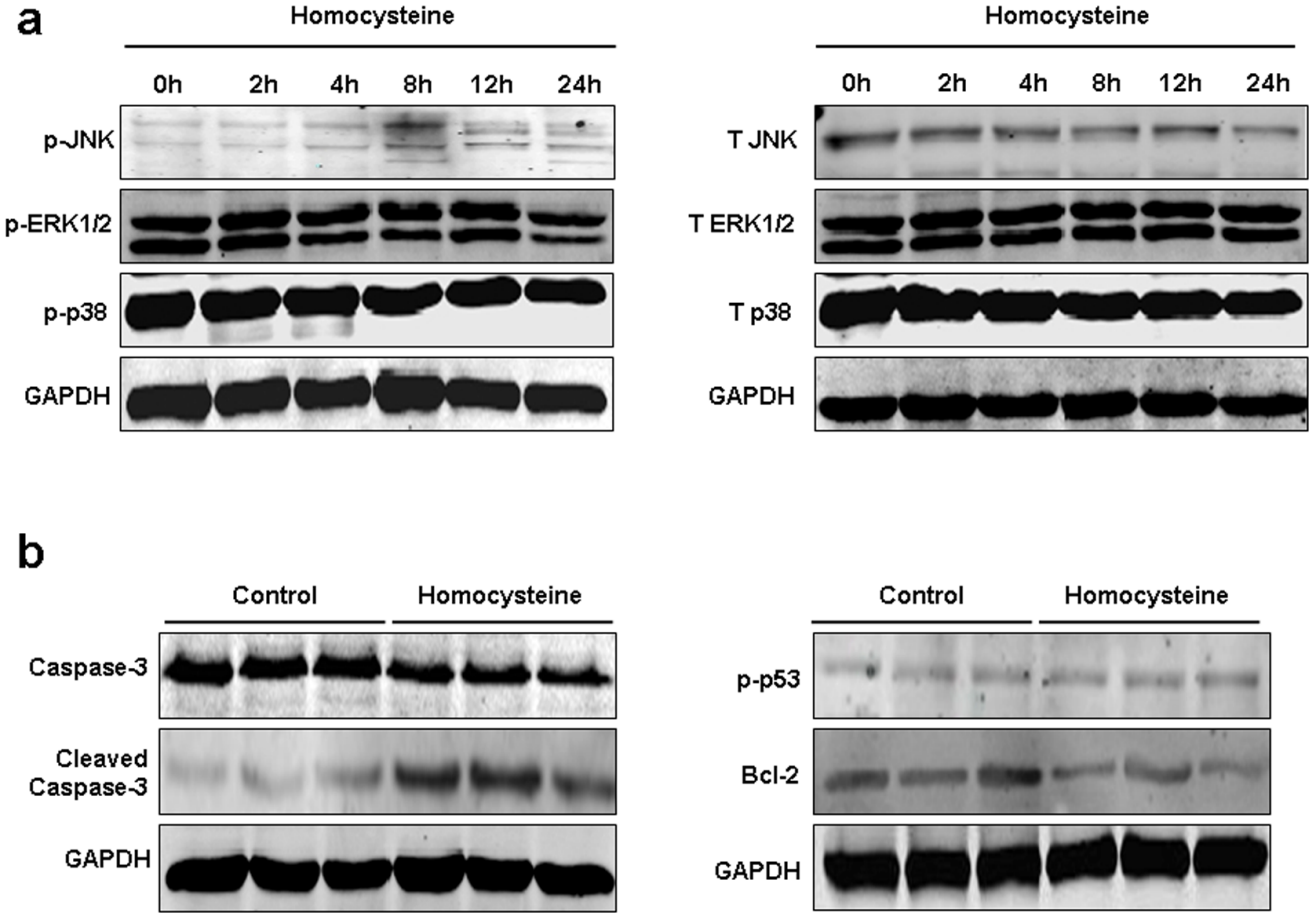
Effects of homocysteine on MAPKs and apoptotic proteins expression in BMSCs. (a) The total and phosphorylated JNK, p38 and ERK1/2 protein was detected by western blotting in BMSCs after treatment with homocysteine at the time point of 0, 2, 4, 8, 12 and 24 h. Homocysteine effectively activated phosphorylated JNK expression after treatment with homocysteine. But homocysteine did not increase the expression of total JNK protein in BMSCs. (b) Influences of homocysteine on the expression of Bcl-2, caspase-3, cleaved caspase-3, and p-p53 proteins in BMSCs. n = 3 independent experiments.

To confirm that JNK pathway contributed to homocysteine-induced BMSCs apoptosis, western blot was utilized to detect the expression of JNK, p38 and ERK1/2, as well as p-p53, caspase-3, cleaved caspase-3, Bcl-2 proteins in BMSCs with or without homocysteine 300 µM treatment. Figure 6a showed that homocysteine 300 µM can increase phosphorylated JNK expression (Figure 6a). Moreover, homocysteine treatment did not significantly alter phosphorylated p38 and ERK1/2 protein expression in BMSCs. In order to confirm that homocysteine induced BMSCs apoptosis, we also detected the expression of p-p53, caspase-3, cleaved caspase-3 and Bcl-2 proteins after homocysteine treatment. As shown in Figure 6b, homocysteine did not impact the expression of p-p53, but increased cleaved caspase-3 expression. Bcl-2 was markedly decreased by homocysteine treatment in BMSCs.

### Homocysteine Reduced VEGF and IGF-1 Released by BMSCs

**Figure 7:**
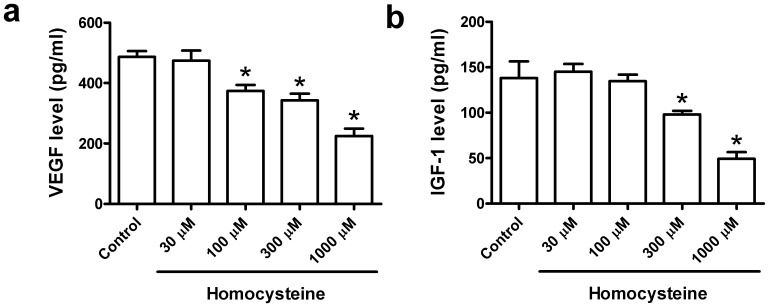
Homocysteine reduced the release of VEGF and IGF-1 by BMSCs. (a) Homocysteine induced a considerable inhibition of VEGF level in culture medium of BMSCs. (b) IGF-1 level was also obviously decreased by homocysteine in BMSCs. * p<0.05 versus Control.

We further explore whether homocysteine treatment leads to the changes of BMSCs functions. The VEGF and IGF-1 levels in the culture medium of BMSCs before and after homocysteine treatment were determined by ELISA assay. Figure 7a showed that homocysteine induced a considerable inhibition of VEGF level in culture medium of BMSCs. Likewise, IGF-1 level was also obviously inhibited by homocysteine in BMSCs (Figure 7b). These suggest that the paracrine function of BMSCs was impaired by homocysteine treatment.

## Discussion

We uncovered for the first time that homocysteine, a novel important independent risk factor for cardiovascular diseases leads to apoptosis of BMSCs via ROS-mediating JNK pathway. Our study provides new insight into the mechanism underlying homocysteine-related BMSCs apoptosis.

BMSCs, not as previously considered, only played a regulatory role in hematopoietic niches [1]–[3]. Recently studies uncovered that BMSCs also have the ability to differentiate into multiple lineages such as cardiomyocytes, endothelial cells, neuron, and adipocytes [4]. More importantly, BMSCs in the bone marrow or peripheral blood was shown to migrate to the heart tissues, and then repair the damaged myocardium by releasing many cellular factors including VEGF-1, IGF-1, etc which may prevent heart against ischemia, oxidant stress, inflammatory injury, and also stimulate cardiac stem cells proliferation and differentiation [27]–[29]. On the contrary, BMSCs dysfunction or apoptosis will exaggerate cardiovascular diseases due to the decreased mobilization and recruitment of BMSCs to injured myocardial tissues [15], [16].

Elevated plasma level of homocysteine has long been known as a new risk factor for cardiovascular diseases [20], [30]. Hyperhomocysteinemia has been shown to cause endothelial dysfunction and apoptosis, promote vascular smooth muscle cell proliferation, increase platelet aggregation and accelerate thrombin formation through free radical formation [31], [32]. Moreover, lots of studies also reported that hyperhomocysteinemia caused the reduction of myocardium contractility, the disruption of cardiac electrical activity, and the apoptosis or necrosis of cardiomyocytes, which is at least partially responsible for its toxic effects on hearts [19], [33]–[36]. In the light of the important role of BMSCs in maintaining and repairing cardiovascular functions, we hypothesized that homocysteine-induced apoptosis of BMSCs serve as a novel mechanism underlying hyperhomocysteinemia-related cardiovascular diseases, and the present study was therefore undertaken to determine whether increased homocysteine level is capable to induce BMSCs apoptosis.

In this study, we uncovered that elevated homocysteine level led to an increase of apoptosis of BMSCs characterized by cellular shrinkage, nuclei condensation and fragmentation, and the formation of apoptotic bodies. Increased apoptosis of BMSCs will subsequently decrease the ability of BMSCs to repair the damaged hearts. Plenty of evidence has confirmed that reactive oxygen species (ROS)-induced oxidative stresses play a key role in the induction of apoptosis under both physiological and pathological conditions [24], [25], [37]. Increased ROS is responsible for the disruption of mitochondrial homeostasis and the depolarization of mitochondrial membrane potential which plays a critical role in maintaining cellular energy and metabolism balance [37]. The dysfunction of the mitochondria will trigger cellular apoptosis by causing the release cytochrome c that triggers caspase activation. In agreement, our study also revealed that exposure to homocysteine can increase intracellular ROS level and in turn cause the depolarization of mitochondrial membrane potential in BMSCs. To determine that ROS is required for homocysteine-induced apoptotic changes of BMSCs, two antioxidants DMTU and NAC were used to inhibit intracellular ROS accumulation induced by homocysteine. The results demonstrated that both DMTU and NAC can reverse the apoptosis of BMSCs induced by homocysteine. In addition, the inhibition of intracellular ROS with antioxidants also attenuated homocysteine-induced depolarization of mitochondrial membrane potential, indicating ROS-mediate mitochondrial damage contributes to the apoptosis of BMSCs.

The MAPK signaling p38 MAPK, JNK and ERK has been positively implicated in the induction of apoptosis in response to oxidant stress signals [26]. Especially, the activated p38 MAPK, JNK and ERK were frequently observed involved in ROS-mediated cellular apoptosis [38]. Recent studies also reported that ROS-mediated activation of p38 and JNK induce the phosphorylation of Bcl-2, which results in mitochondrial apoptotic cell death [39]. In this study, we further investigated the role of MAPK signaling in ROS-mediated mitochondrial apoptotic cell death triggered by homocysteine. The results showed that the blockage of JNK with its specific inhibitor can abrogate homocysteine-induced mitochondrial apoptotic cell death, but p38 MAPK and ERK specific inhibitors did not impact homocysteine-induced apoptosis of BMSCs. It suggests that the activation of JNK is involved in homocysteine-induced apoptotic morphological changes. We also detected the expression of caspase-3, p53 and Bcl-2 to confirm if homocysteine leads to the apoptosis of BMSCs. The results showed that homocysteine treatment caused an increase of cleave caspase-3 protein and decrease of Bcl-2 protein in BMSCs, indicating the proapoptotic role of homocysteine in BMSCs.

The concentration of homocysteine that we used in the cultured cells is higher than plasma homocysteine level under physiological condition, which can not be avoided since the metabolism of homocysteine was considerably upregulated in the cells in culture as described in previous studies [40]. Actually, the same or higher level of homocysteine has been widely used in a variety of previous investigations [41]–[43]. Moreover, a high concentration of homocysteine is required to mimics the long-term effects of slight or middle increase of homocysteine in human bodies.

### Conclusion

Taken together, we uncovered that increased homocysteine level enhanced intracellular ROS production and caused the depolarization of mitochondrial membrane potential, and in turn led to the apoptosis of BMSCs via activating JNK signal. These findings lead to a better understanding of the molecular mechanism of hyperhomocysteinemia-associated cardiovascular diseases.
